# Infectious Bronchitis Virus Infection Induces Apoptosis during Replication in Chicken Macrophage HD11 Cells

**DOI:** 10.3390/v9080198

**Published:** 2017-07-26

**Authors:** Xiaoxiao Han, Yiming Tian, Ru Guan, Wenqian Gao, Xin Yang, Long Zhou, Hongning Wang

**Affiliations:** 1 Key Laboratory of Bio-Resources and Eco-Environment, Ministry of Education, College of Life Science, Sichuan University, Chengdu 610064, China; silky1010@163.com (X.H.); tymgood@126.com (Y.T.); 17713568487@163.com (R.G.); 18780204364@163.com (W.G.); yangxin0822@163.com (X.Y.); wszl5918@163.com (L.Z.); 2 Animal Disease Prevention and Food Safety Key Laboratory of Sichuan Province, Chengdu 610064, China; 3 “985 Project” Science Innovative Platform for Resource and Environment Protection of Southwestern China, Sichuan University, Chengdu 610064, China

**Keywords:** IBV infection, chicken macrophage, apoptosis, caspase, virus replication

## Abstract

Avian infectious bronchitis has caused huge economic losses in the poultry industry. Previous studies have reported that infectious bronchitis virus (IBV) infection can produce cytopathic effects (CPE) and apoptosis in some mammalian cells and primary cells. However, there is little research on IBV-induced immune cell apoptosis. In this study, chicken macrophage HD11 cells were established as a cellular model that is permissive to IBV infection. Then, IBV-induced apoptosis was observed through a cell viability assay, morphological changes, and flow cytometry. The activity of caspases, the inhibitory efficacy of caspase-inhibitors and the expression of apoptotic genes further suggested the activation of apoptosis through both intrinsic and extrinsic pathways in IBV-infected HD11 cells. Additionally, ammonium chloride (NH_4_Cl) pretreated HD11 cells blocked IBV from entering cells and inhibited IBV-induced apoptosis. UV-inactivated IBV also lost the ability of apoptosis induction. IBV replication was increased by blocking caspase activation. This study presents a chicken macrophage cell line that will enable further analysis of IBV infection and offers novel insights into the mechanisms of IBV-induced apoptosis in immune cells.

## 1. Introduction

Infectious bronchitis virus (IBV) can cause avian infectious bronchitis, an acute and highly infectious disease of chicken. IBV is a member of the family *Coronaviridae* and genus *Coronavirus*. It is a single stranded positive sense, enveloped RNA virus 27–32 kb in length [1,2]. Like some other members of the coronavirus family, IBV mainly causes upper-respiratory tract disease. IBV is characterized by nephritis, proventriculitis and reduction in both laying rate and egg quality in infected chickens [3]. Vaccination is an effective prevention measure, but IBV’s ability to mutate has decreased vaccine protection [4,5,6]. In order to develop better prevention and control measures, the interactions between host and IBV needs to be better studied. Almost all wild-type IBV strains are only able to proliferate in embryonated chicken eggs or primary chicken embryo kidney cells. The Beaudette strains were used previously to study the resistance of IBV to the antiviral state induced by type I interferon (IFN) [7], induction of apoptosis through endoplasmic reticulum stress in Vero cells by IBV infection [8] and activate autophagy by IBV nonstructural protein (NSP) 6 [9]. However, there have been limited studies of the interactions between IBV infection and immune cell apoptosis. Here, the Beaudette strain was used to study the mechanism of IBV infection.

Apoptosis is a form of programmed cell death that results from the activation of intracellular self-destruction biochemical programs [10]. The activation of caspases (a family of cysteine protease) is a significant regulatory event in the apoptosis process [11]. Caspase cascades are triggered by both extrinsic and intrinsic signals to mediate the cell apoptosis [12]. The relationship between cell apoptosis and virus infection is complex. Cell apoptosis induced by virus may cause tissue damage, especially in the immune and nervous systems, suggesting that apoptosis is a pathogenic mechanism in virus-induced disease. At the same time, apoptosis of infected cells can directly interfere with viral replication, and immune cells can engulf apoptotic cells to prevent inflammation [13,14].

Previous studies demonstrated that IBV induced apoptosis in cultured mammalian cells and primary cells [6,15,16]. However, there is limited information about the apoptosis signaling pathways induced by IBV infection in immune cells. Some studies indicated that IBV can transform certain elements of the innate immune system to promote secondary bacterial infections, and macrophages, as an important component of the innate immune system, may play a role in this process [17]. A nephropathogenic IBV strain (B1648) can productively replicate in blood monocytic cells, and the infected cells may act as carrier cells to play a crucial role in cell-associated viremia and the dissemination of virus to the internal organs [18]. Some viruses have been shown to induce apoptosis in macrophage, like human immunodeficiency virus (HIV)-1 [19], Chikungunya virus (CHIKV) [20] and influenza virus [21]. Therefore, additional study is required to investigate the functional roles of macrophages in IBV infection to help understand the mechanistic details of immune responses during virus infections [22].

In this report, we used chicken macrophage HD11 cells considered an accurate representation of primary avian macrophages [23,24]. The HD11 cells were identified and characterized as a novel model that is permissive to IBV infection. The molecular and morphological variations in IBV-infected cells revealed that cell apoptosis was induced by IBV infection and appeared to activate caspase-8 by the Fas/Fas ligand (FasL)-mediated signaling pathway and to activate caspase-9 by the B-cell lymphoma 2 (Bcl-2) family-mediated signaling pathway. Apoptosis required viral replication in IBV-infected cells.

## 2. Materials and Methods

### 2.1. Cells and Virus

The chicken macrophage HD11 cells were kindly provided by Prof. Xin-An Jiao (Jiangsu Key Laboratory of Zoonosis, Yang Zhou University, Yang Zhou, Jiangsu Province, China). HD11 cells were cultured in Dulbecco’s modified Eagle’s medium (DMEM) (HyClone, Logan, UT, USA) supplemented with 10% fetal bovine serum (FBS) (Gemini Bio-Products, West Sacramento, CA, USA), 100 U/mL penicillin and 100 μg/mL streptomycin (HyClone, Logan, UT, USA) at pH 7.2 and were kept at 37 °C with 5% CO_2_. The Vero cell-adapted IBV Beaudette strain (p65) [25] used in the current study was kindly provided by Prof. Shi-Qi Sun (State Key Laboratory of Veterinary Etiological Biology, Lanzhou Veterinary Research Institute, Chinese Academy of Agricultural Sciences, Gansu Province, China). Traditional IBV strain M41, vaccine IBV strain H120 and virulent IBV strain SABIK2 [26] were housed in our laboratory (viruses were propagated in specific pathogen free (SPF) 10 days old embryonated chicken eggs). Susceptibility of HD11 cells to IBVs was measured by morphological changes, growth curves using 50% tissue culture infective doses (TCID_50_) and indirect immunofluorescence assay (IFA).

### 2.2. Virus Titration and Growth Kinetics

Here, TCID_50_ were applied to HD11 cells to quantitate virus titers as described previously [27]. HD11 cells were cultured in 96-well plates, and ten-fold dilutions of the virus were prepared in DMEM supplemented with 2% FBS. Cultured cells were infected with the virus and then observed daily for cytopathic effects (CPE). In order to evaluate the virus growth kinetics in HD11 cells, the cells were infected with IBV at 10 multiplicity of infection (MOI). The infected cells were collected at the indicated time points and analyzed using TCID_50_ assay.

### 2.3. Cell Viability Assay

A Cell Counting Kit-8 (CCK-8) assay (Beyotime, Haimen, Jiangsu Province, China) was utilized to identify the viability of cells as described previously [28]. HD11 cells were cultured in 96-well plates and infected with IBV at different MOI (0.1, 0.5, 1, 5, and 10 MOI) for specific lengths of times. In parallel, a negative control was set up. The cells were incubated with 10 μL/well CCK-8 solution (Beyotime, Haimen, Jiangsu Province, China) and allowed to react for 2 h at 37 °C. The absorbance was detected using a microplate reader (model 680, Bio-Rad, Hercules, CA, USA) at 450 nm. The negative control was set at 100%, and the treated samples were calculated according to the following formula: Survival rate (%) = optical density (OD) of the treated cells/OD of the negative control × 100.

### 2.4. Morphological Analysis

HD11 cells were pre-incubated in 96-well plates and infected with IBV at 10 MOI. To assess apoptosis, the condensed and fragmented nuclei were observed using Hoechst 33342 staining (KeyGEN Biotech, Nanjing, Jiangsu Province, China). At the specified time points, the cells were immobilized with 4% paraformaldehyde (KeyGEN Biotech, Nanjing, Jiangsu Province, China) for 30 min and then incubated with Hoechst 33342 (KeyGEN Biotech) in the dark for 15 min. The typical apoptotic morphological changes were observed using a fluorescence microscope (Olympus IX71, Olympus, Tokyo, Japan) with UV excitation at 350 nm.

### 2.5. Indirect Immunofluorescence Assay

HD11 cells were grown overnight to 75% density in 96-well plates and were then infected with IBV at an MOI of 10. After the appearance of typical CPE, the cells were immobilized with 4% paraformaldehyde for 30 min. A mouse polyclonal antibody against the IBV nucleocapsid (N) protein (1:200 dilution, prepared in our laboratory) was added, followed by incubation for 1 h at 37 °C. Next, the cells were treated with a fluorescein isothiocyanate (FITC)-conjugated goat anti-mouse IgG (1:200, TransGen Biotech, Beijing, China) for 1 h at 37 °C. The specimens were viewed with an Olympus IX71 fluorescence microscope (Olympus) with the appropriate excitation and emission wavelengths for FITC (490 nm and 525 nm, respectively).

### 2.6. Flow Cytometry

To identify the apoptotic rate, the percentage of cells undergoing apoptosis was determined by an annexin V-FITC apoptosis detection kit (Absin, Shanghai, China). HD11 cells were cultured in 6-well plates and infected with IBV at 10 MOI. Cells were harvested and washed three times with phosphate-buffered saline (PBS) at the indicated times. The cells were centrifuged at 500 g for 5 min and then suspended in 500 μL of binding buffer containing 5 μL FITC-conjugated annexin V antibody and 5 μL propidium iodide (PI). The mixture was incubated at room temperature for 15 min in the dark. The cells were detected by flow cytometer (BD Biosciences, San Jose, CA, USA) within an hour.

### 2.7. Caspase Activity Assay

The activities of caspase-3, -8, and -9 were detected by colorimetric assay kit (KeyGEN Biotech). The cells were incubated with lysis buffer, and the concentrations of protein were detected by bicinchoninic acid (BCA) protein assay reagent (Vazyme Biotech, Nanjing, Jiangsu Province, China). The protein (200 μg/sample) was treated with caspase-3, -8, and -9 substrate for each sample at 37 °C for 4 h. Samples were read by a microplate reader (model 680, Bio-Rad) at 405 nm.

### 2.8. Quantitative Real-Time Polymerase Chain Reaction Analysis

Total RNA was isolated using TRIzol agent (Invitrogen, Carlsbad, CA, USA), and each RNA sample was reverse-transcribed to complementary DNA (cDNA) by PrimeScript™ RT Reagent Kit (Takara, Dalian, Liaoning Province, China). cDNA was used for quantitative real-time polymerase chain reaction (qRT-PCR) analysis. The sets of primer pairs of apoptotic regulating genes are listed in Table 1 [29]. For qRT-PCR reactions, the 25 μL reaction mixture included 2 μL cDNA, 12.5 μL SYBR Premix Ex TaqTM Ⅱ (Takara), 1.0 μL of forward and 1.0 μL of reverse primer and 8.5 μL RNAase-free water (Takara). Reaction conditions were 95 °C for 3 min followed by 44 cycles of 95 °C for 10 s, the specific melting temperature (Tm) of a primer pair for 30 s, and then 95 °C for 10 s, and 72 °C for 10 s, using a Bio-Rad IQ5 Thermal Cycler (Bio-Rad). β-actin was selected as a reference gene. The expression fold changes were calculated using the 2^−ΔΔCT^ method [30]. 

### 2.9. Statistical Analysis

All data are expressed as the mean ± standard error of the mean (SEM) from three independent experiments performed in triplicate. The statistical analyses were conducted using Student’s *t*-test in GraphPad Prism version 5 (GraphPad Software, San Diego, CA, USA). A *p*-value < 0.05 was considered significant, and a *p*-value < 0.01 was considered highly significant.

## 3. Results

### 3.1. Viral Infection in HD11 Cells

To determine whether IBVs replicate in the chicken macrophage cell line, four IBV strains (Beaudette, M41, H120 and SABIK2 strains) were utilized in this study to test the infective processes in HD11 cells. For the M41, H120 and SABIK2 strains, the infected cells were blindly passaged five times, and CPE was not observed. The growth curve using TCID_50_ and IFA showed that replication of the three IBV strains in HD11 cells was severely restricted, and no significant replication was observed. However, the HD11 cells were highly permissive for the propagation of the attenuated IBV Beaudette strain. The results showed that HD11 cells could be infected by IBV Beaudette in passage one. 

**Figure 1:**
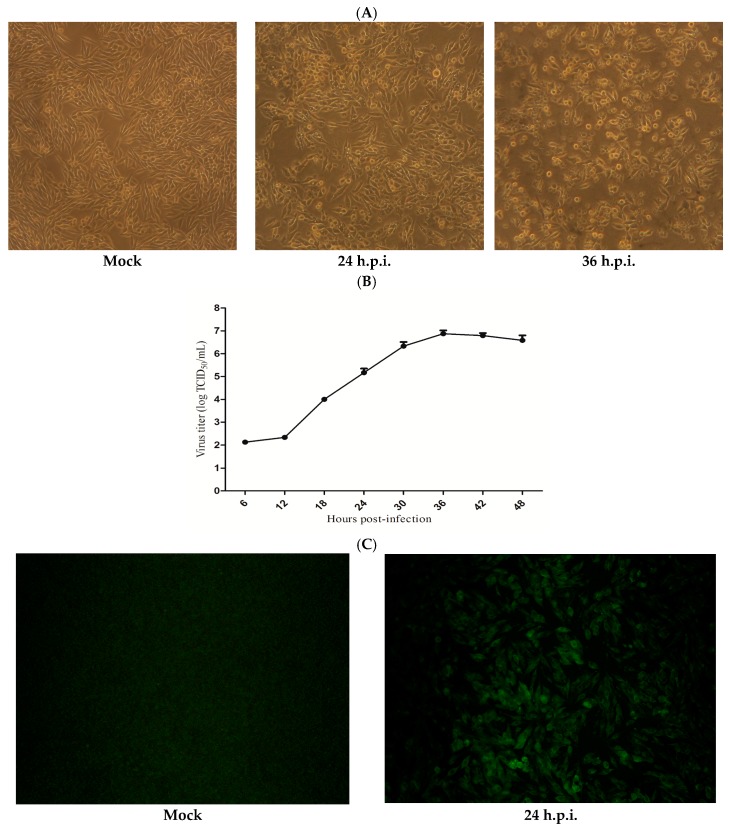
Susceptibility of HD11 to infectious bronchitis virus (IBV) Beaudette infection. (**A**) Cytopathic effects (CPE) observed upon IBV Beaudette infection by 24 and 36 h post-infection (h.p.i.), and mock-infected HD11 cells displayed no CPE. (**B**) Growth kinetics of IBV Beaudette in HD11 cells infected at 10 multiplicity of infection (MOI). Data are shown as the mean ± standard error of the mean (SEM) of three independent experiments. (**C**) Production of IBV Beaudette (fluorescein isothiocyanate, FITC) could be observed at 24 h.p.i., and mock-infected HD11 cells showed no fluorescence.

First, morphological changes of IBV Beaudette-infected HD11 cells were observed. After infection with IBV Beaudette at 10 MOI, CPE appeared in HD11 cells at 24 h post-infection (h.p.i.) and were evident at 36 h.p.i. when compared with the mock infection (Figure 1A). The normal HD11 cells could also be re-infected with the culture supernatant from the virally infected cells. The susceptibility of HD11 cells to IBV Beaudette infection was evaluated by growth kinetics. The growth kinetics of the virus were observed upon infection at an MOI of 10 in HD11 cells. The virus titers increased until reaching the maximal level of 10^6.875^ TCID_50_/mL (Figure 1B). IBV Beaudette replication in HD11 cells was also studied by performing an immunofluorescence assay. The production of FITC-stained virus was observed by 24 h.p.i. In contrast, mock-infected HD11 cells showed no fluorescence (Figure 1C).

### 3.2. IBV Beaudette Induces Apoptosis in HD11 Cells

**Figure 2:**
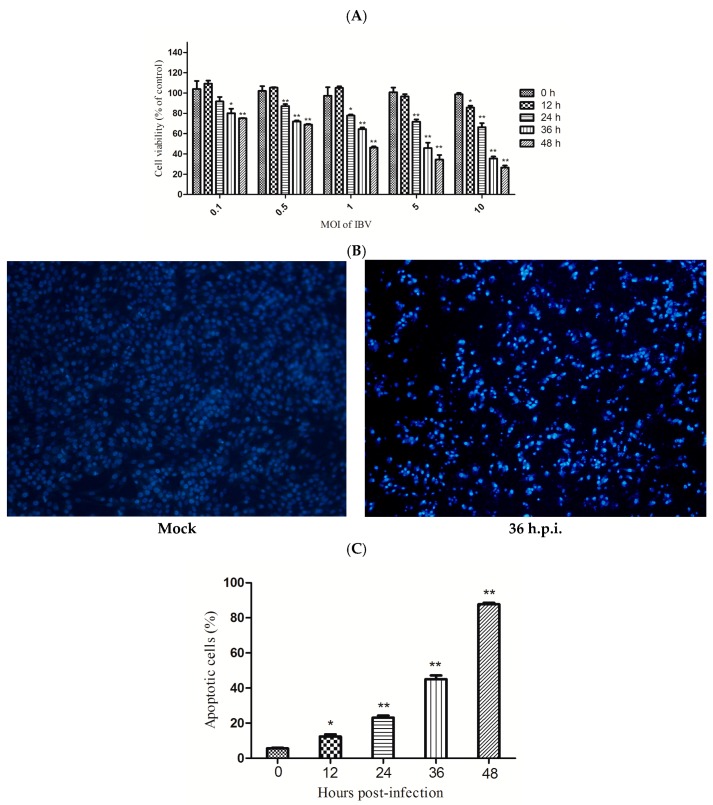
IBV Beaudette induces apoptosis in HD11 cells. (**A**) The role of IBV Beaudette in cell viability. HD11 cells were infected with IBV Beaudette at different MOIs and detected at the indicated times. The Cell Counting Kit-8 (CCK-8) assay was used to measure cell viability. The data are shown as the mean ± SEM of three independent experiments. * *p* < 0.05, ** *p* < 0.01 versus control group (0 h). (**B**) Morphological changes. IBV Beaudette-infected cells were observed with condensed chromatin and nuclear fragmentation under fluorescence microscopy followed by Hoechst 33342 staining. (**C**) The apoptotic rate of cells. IBV Beaudette-infected cells (10 MOI) were subjected to flow cytometry at different times. Data are shown as the mean ± SEM of three independent experiments. * *p* < 0.05, ** *p* < 0.01 versus control group (0 h).

Infection of HD11 cells with IBV Beaudette caused cell death in a time- and dose- dependent manner, as tested by CCK-8 assay. (Figure 2A). The infected cells showed chromatin condensation and nuclear fragmentation. After 36 h of infection, large amounts of apoptotic bodies were observed in HD11 cells (Figure 2B). The rate of apoptotic cells was measured by flow cytometry. The rate of apoptosis significantly increased at 12 h.p.i. in virus-infected cells when compared with the mock-infected cells (Figure 2C). These findings indicated that apoptosis was induced by IBV Beaudette infection in HD11 cells.

### 3.3. IBV Beaudette Triggers Apoptosis by Induction of Caspase Activity

**Figure 3:**
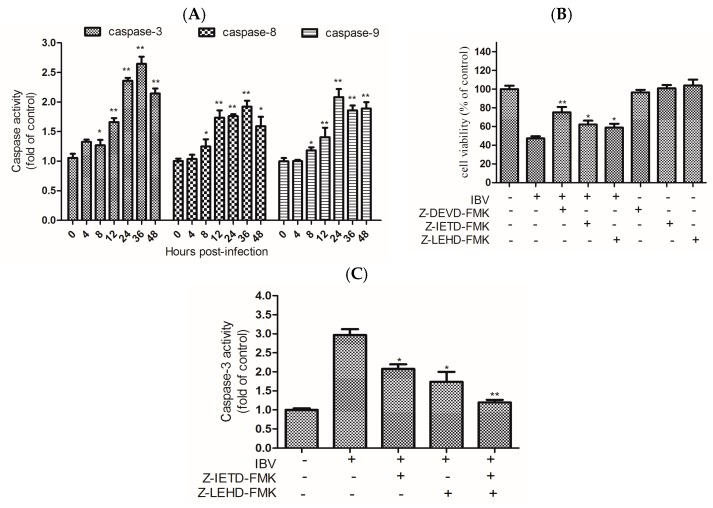
Effects of IBV Beaudette infection on caspases in HD11 cells. (**A**) The activity of caspases in IBV Beaudette-infected cells. The caspases -3, -8 and -9 activity in HD11 cells infected with IBV at 10 MOI at the designed times were determined. The data are shown as the mean ± SEM, * *p* < 0.05, ** *p* < 0.01 versus the control group (0 h). (**B**) Role of caspase inhibitors in cell viability. Cell viability was determined by CCK-8 assay: 20 μM of each caspase inhibitor was utilized to pre-treat cells for 2 h. Then, the treated and untreated cells were both infected with IBV Beaudette at an 10 MOI for 36 h. The data are shown as the mean ± SEM, * *p* < 0.05, ** *p* < 0.01 versus IBV infection alone. (**C**) The effect of initiator caspase-8 or -9 on the activation of caspase-3: 20 μM of each caspase inhibitor was utilized to pretreat cells for 2 h. Then, the treated and untreated cells were infected with IBV at 10 MOI for 36 h. Caspase-3 activity was detected using a colorimetric assay kit. Data are shown as the mean ± SEM, * *p* < 0.05, ** *p* < 0.01 versus virus infection alone.

Activation of the caspase proteinases is a significant event in the occurrence of apoptosis. The activity of caspases that play important roles in the activation of the apoptosis pathway was investigated in this study. When HD11 cells were infected with IBV Beaudette at 10 MOI, the levels of caspase-3, -8, and -9 were significantly increased from 8 h.p.i. and then increased further over time (Figure 3A). To further identify the function of caspase-3, -8, and -9 in the apoptotic pathway, we measured the viability of infected-cells incubated with specific inhibitors of caspase-3, -8, and -9 (Z-DEVD-FMK, Z-IETD-FMK, and Z-LEHD-FMK; KeyGEN Biotech, Nanjing, Jiangsu Province, China). The data revealed that cell viability was significantly increased by the specific inhibition of caspase-3, -8, and -9 (Figure 3B). To confirm the function of caspase-8 and caspase-9 to activate caspase-3, the inhibitory efficacy of the caspase-8 or caspase-9 inhibitors on caspase-3 activity was also determined. When cells were pretreated with the caspase-8 or caspase-9 inhibitor, the activity of caspase-3 was significantly decreased in cells, and more significantly decreased when the two inhibitors were added together (Figure 3C). These results revealed that caspase-3 activation and IBV Beaudette-induced apoptosis may be triggered via both extrinsic and intrinsic pathways.

### 3.4. Regulation of IBV Beaudette-Inducted Apoptosis Is Regulated by the Fas/FasL and Bcl-2 Family Members

**Figure 4:**
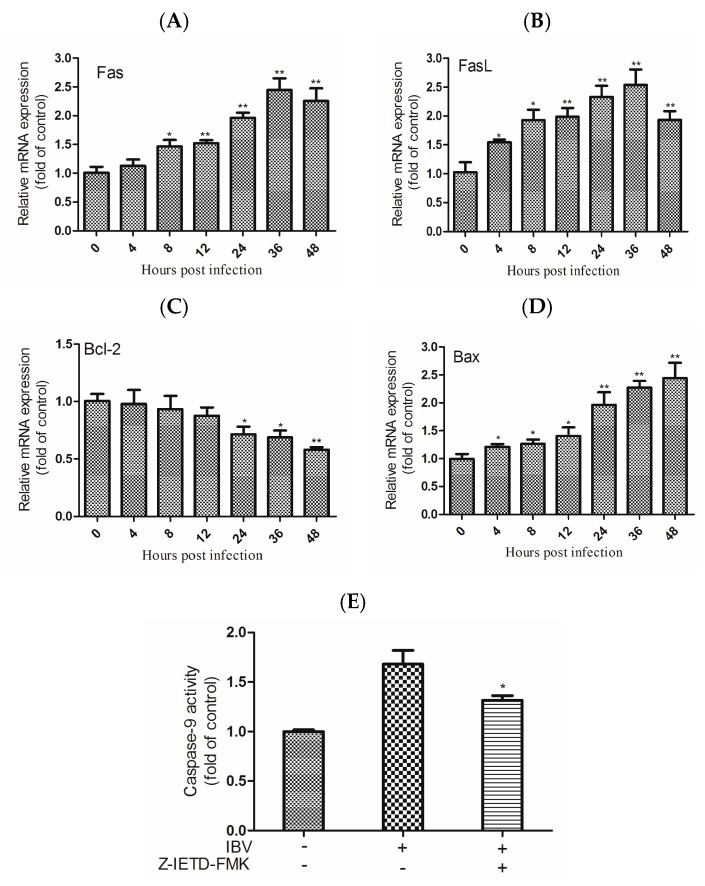
IBV Beaudette-induced apoptosis was regulated by the expression of Fas/Fas ligand (FasL) and the Bcl-2 family. (**A**,**B**) mRNA expression levels of Fas and FasL were detected in IBV Beaudette-infected cells for the indicated times. The data are shown as the mean ± SEM. * *p* < 0.05, ** *p* < 0.01 versus control. (**C**,**D**) mRNA expression levels of Bcl-2 and Bax were detected in IBV Beaudette-infected cells for indicated times. The data are shown as the mean ± SEM. * *p* < 0.05, ** *p* < 0.01 versus control. (**E**) The activity of caspase-9 was party blocked by caspase-8 inhibitor. Following incubation with Z-IETD-FMK for 2 h, the cells were infected with IBV Beaudette for 24 h. Caspase-9 activity was detected using a colorimetric assay kit. Data are shown as the mean ± SEM. * *p* < 0.05 versus virus infection alone.

Caspase-8 has an important effect on apoptosis that is mediated by Fas/FasL. The activity of caspase-8 was increased in the IBV Beaudette-infected HD11 cells. This implied that apoptosis is induced by IBV Beaudette infection through the Fas/FasL pathway. To investigate this further, the expression levels of Fas and FasL were detected in IBV Beaudette-infected HD11 cells by qRT-PCR. The data revealed increased gene expression of Fas and FasL over time (Figure 4A). Furthermore, the members of the Bcl-2 family are generally distributed on the surface of mitochondria, and their activation may regulate the intrinsic apoptosis pathway. To test this, the expression levels of Bcl-2 and Bcl-2- associated X protein (Bax) were quantified by qRT-PCR in IBV Beaudette-infected HD11 cells. The results showed the mRNA levels of Bcl-2 were obviously downregulated from 24 h.p.i. and declined over time. Conversely, the mRNA levels of Bax were upregulated from 4 h.p.i. and continuously increased until 48 h.p.i (Figure 4B). Moreover, the activation of caspase-9 was partly inhibited in IBV Beaudette-infected cells pretreated with the inhibitor of caspase-8 (Figure 4C). This suggested that caspase-9 activation was affected by the blocking of caspase-8 activity. Taken together, these findings suggested that the Fas/FasL-mediated signal contributes to the activation of caspase-8. Additionally, Bcl-2 and Bax might play important roles in regulating the activation of caspase-9. The activation of caspase-8 can also affect the extrinsic apoptosis pathway in IBV Beaudette-infected cells.

### 3.5. Cell Apoptosis and Viral Replication are Required Mutually

**Figure 5:**
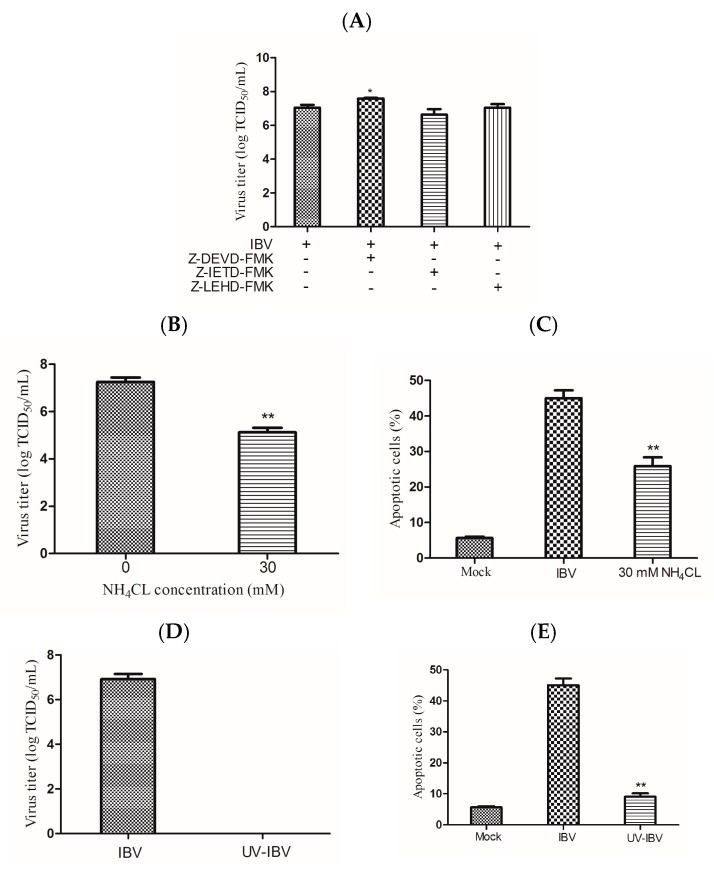
Interplay between cell apoptosis and virus replication. (**A**) Following incubation with inhibitors for 2 h, the cells were infected with IBV Beaudette of 10 MOI for 36 h. After pre-treatment, virus titers were determined as log TCID_50_/mL. Data are shown as the mean ± SEM. * *p* < 0.05 versus virus infection alone. (**B**,**C**) A 30 μM concentration of NH_4_Cl was used to incubate HD11 cells for 2 h before infection, then cells were infected with IBV Beaudette of 10 MOI for 36 h, and the virus titer (**B**) and the rate of apoptotic cells (**C**) were separately tested. Data are shown as the mean ± SEM. ** *p* < 0.01 versus cells infected without NH_4_Cl. (**D**,**E**) UV germicidal light was utilized to inactivate IBV Beaudette for 30 min. UV-inactivated virus infected HD11 cells for 36 h; the virus titer (**D**) and the rate of apoptotic cells (**E**) were measured separately. Data are shown as the mean ± SEM. ** *p* < 0.01 versus UV-untreated IBV Beaudette.

To determine whether apoptosis plays a pivotal role in inhibition of virus replication, the virus titers of untreated cells or those treated with caspase inhibitors were detected by TCID_50_. The results showed that the caspase-3 inhibitor could increase the titer of IBV Beaudette, but did not show obvious effects on caspase-8 and -9 inhibitor-treated cells (Figure 5A). To test whether the ability of virions to enter cells was crucial to apoptosis, endosomal acidification was blocked by NH_4_Cl to prevent the viruses from being released [31]. The virus titer was significantly decreased with NH_4_Cl treatment (Figure 5B). Compare with non-treated cells, the rate of apoptotic cells was also decreased in NH_4_Cl-treated cells when infected with IBV Beaudette (Figure 5C). Next, the UV-treated virus was used to test whether apoptosis induction required virus replication. When the virus was subjected to UV treatment, the virus titer could not be detected (Figure 5D). Consistently, the rate of apoptotic cells was dramatically decreased in cells infected with UV-inactivated virus, when compared with the UV-untreated virus (Figure 5E). In conclusion, apoptosis induction required viral replication in IBV Beaudette-infected cells.

## 4. Discussion

Compared with other coronaviruses, IBV is not easily adapted to cell culture. Several mammalian cell lines and primary cells have been previously revealed to be permissive to IBV infection. Some strains of IBV can replicate and produce CPE in primary chicken embryo kidney cells. IBV Holte and Beaudette-42 strains can proliferate in the BHK-21 cell line [32], and the Beaudette strain of embryo-culture IBV has adapted to Vero cells [33]. However, previous studies of chicken immune cells and the pathogenesis of IBV focused on primary immune cells separated from the blood or spleen [18,34]. In this study, HD11 cells, a chicken macrophage cell line, were shown to be susceptible to IBV Beaudette. Additionally, the IBV Beaudette-infected cells produced infectious virus progeny with a high virus titer. Morphological assessment of the cells during IBV Beaudette infection showed that CPE can be observed after 24 h.p.i. The virus growth kinetics for HD11 cells also showed peak viral titers at 36 h.p.i. Immunofluorescence was used to identify and analyze virus infection, and strong fluorescence signals were observed in the IBV Beaudette-infected cells. Based on these results, the chicken macrophage HD11 cells will serve as an essential tool for future studies of IBV infection.

Apoptosis is an important part of the antiviral host response. However, some viruses actively trigger this process to facilitate their replication [13]. Infection with coronavirus induced apoptosis in various cell types. Transmissible gastroenteritis virus (TGEV)-induced apoptosis in PK-15 cells was dependent on viral replication [31]. Porcine hemagglutinating encephalomyelitis virus (PHEV) induced apoptosis through a caspase-dependent pathway in PK-15 cells [35]. Severe acute respiratory syndrome (SARS) coronavirus membrane (M) and nucleocapsid (N) proteins can induce apoptosis in HPF cells [36]. According to previous studies, apoptosis occurs in response to IBV infection in Vero cells, DF1 cells and chicken embryo kidney cells [16,37]. This study is the first demonstration that IBV induces apoptosis in chicken macrophage HD11 cells. The IBV Beaudette-infected HD11 cells exhibited typical characteristics of apoptosis including the condensation of the cell nucleus, reduction of cell viability, and an increased rate of apoptotic cells.

Caspases are a family of cysteine-catalyzed proteases that cleave aspartic acids. The triggering of caspase cascades plays indispensable roles in apoptosis and can be induced by many viruses. There are two major signaling pathways that contribute to caspase activation: death receptor and mitochondrial pathways [38]. Some viruses have exhibited cell apoptosis that is mediated by Fas/FasL signaling as a reaction to viral infection, such as hepatitis C virus (HCV) [39] and dengue virus (DENV) [40]. Cell apoptosis can be induced by some viruses by regulating the levels of Bcl-2 family members, such as SARS coronavirus [41] and Epstein–Barr virus (EBV) [42]. Our results showed that the activation of caspase-8 in IBV Beaudette-infected cells was regulated by Fas and FasL. The results also showed that activation of caspase-9 in IBV Beaudette-infected cells was regulated by decreased expression of Bcl-2 and increased expression of Bax. The caspase-3 activation and virus-induced apoptosis might be triggered through both extrinsic and intrinsic pathways. In most cases, cell apoptosis induced by virus is a process of interaction between extrinsic and intrinsic pathways. The activation of caspase-8 was inhibited by Z-IETD-FMK, and the activation of caspase-9 was not completely eliminating by blocking caspase-8 activity, suggesting that the activation of caspase-8 is not the only pathway to activate caspase-9, requiring further research. IBV are known to induce apoptosis through caspase-dependent pathway [15] and intrinsic-dependent pathway regulated by Bcl-2 family proteins [16] in Vero cells. In this study, these two pathways were demonstrated that play an important role in IBV Beaudette-infected HD11 cells. Additionally, this is the first report that the extrinsic pathway regulated by Fas/FasL was activated in IBV-induced apoptosis. Improved knowledge of the mechanisms by which IBV activates the extrinsic and intrinsic apoptotic pathways will help to better understand the pathogenic properties of epidemic IBV strains in the host.

Some viruses can induce cell apoptosis through viral replication. UV-inactivated BHV-1 and TGEV could not induce apoptosis, or the NH_4_Cl-pretreated cells prevented the appearance of apoptosis [31,43]. Some other viruses can induce apoptosis without viral replication, such as vaccinia virus and vesicular stomatitis virus [44,45]. Here, infection with UV-inactivated IBV Beaudette or treatment of HD11 cells with NH_4_Cl reduced virus apoptosis induction, indicating that IBV Beaudette -induced apoptosis in HD11 cells depends on viral replication. This finding is similar to that of a previous study showing that UV-inactivated IBV lost the capacity to induce apoptosis in mammalian cells [16]. We tested whether caspase activation is needed for IBV replication in cells. The finding revealed that treatment with the caspase-3 inhibitor can increase the virus titer in IBV Beaudette-infected cells, suggesting that the caspase inhibitor might increase the survival time of cells to promote replication. From a therapeutic standpoint, available drugs controlling apoptosis could be used to limit IBV spreading [46].

The innate immune response is the first line of defense against viruses, and macrophages are an important component of this system. Some viruses have evolved strategies to induce apoptosis to enhance the production of virus progeny and promote dissemination to neighboring cells with limited host immune/inflammatory responses. The presence of apoptotic cells may also lead to the mobilization and initiation of innate immune defenses [47]. Previous studies have shown that virus-induced apoptosis of macrophage has an important impact on virus infection. Porcine reproductive and respiratory syndrome virus (PRRSV) stimulates anti-apoptotic pathways in macrophages early in infection, and these PRRSV-infected macrophages die by apoptosis late in infection [48]. CHIKV infection induces apoptosis and enhances expression of major histocompatibility complexes (MHCs) and co-stimulatory molecules and interleukin (IL)-6 and monocyte chemoattractant protein (MCP)-1 production in macrophages [20]. However, little is known about IBV-induced immune cell apoptosis. It has been reported that phagocytic cells may play a crucial role in dissemination of virus to the blood circulation and internal organs. Therefore, establishment of this macrophage system of IBV Beaudette infection and determination of the apoptotic mechanism might be proof of principle for IBV infection in the host.

In conclusion, chicken macrophage HD11 cells were established for attenuated IBV strain Beaudette infection. IBV Beaudette induced cell apoptosis through caspase-8 activation mediated by Fas/FasL and caspase-9 activation mediated by Bcl-2/Bax. In addition, IBV Beaudette replication was essential to apoptosis induction, and IBV Beaudette replication increased when caspase activation was blocked. Based on these findings, this study has shown the establishment of a chicken macrophage cell line that will facilitate the further analysis of IBV infection. Additional studies are required to clarify the detailed molecular mechanisms underlying IBV-induced apoptosis.

## Figures and Tables

**Table 1:** Sequences of chicken primer pairs used for quantitative real-time polymerase chain reaction (qRT-PCR).

Gene	Forward Primer (5′–3′)	Reverse Primer (5′–3′)
Fas	TCCACCTGCTCCTCGTCATT	GTGCAGTGTGTGTGGGAACT
FasL	GGCATTCAGTACCGTGACCA	CCGGAAGAGCACATTGGAGT
Bax	GGTGACAGGGATCGTCACAG	TAGGCCAGGAACAGGGTGAA
Bcl-2	TGTTTCTCAAACCAGACACCAA	CAGTAGGCACCTGTGAGATCG
β-actin	TGCTGTGTTCCCATCTATCG	TTGGTGACAATACCGTGTTCA
